# Multicompartment Ecosystem Mass Balances as a Tool for Understanding and Managing the Biogeochemical Cycles of Human Ecosystems

**DOI:** 10.1100/tsw.2001.98

**Published:** 2001-10-09

**Authors:** Lawrence A. Baker, Diane Hope, Ying Xu, Jennifer Edmonds

**Affiliations:** Baker Environmental Consulting, Mounds View, MN 55112, USA

**Keywords:** nitrogen pollution, nitrogen cycling, biogeochemical cycles, mass balances, nutrient cycling, groundwater, wastewater treatment

## Abstract

Nitrogen remains a ubiquitous pollutant in surface and groundwater throughout the United States, despite 30 years of pollution control efforts. A detailed multicompartment N balance for the Central Arizona-Phoenix ecosystem is used to illustrate how an ecosystem-level approach can be used to develop improved N management strategies. The N balance is used to demonstrate how nitrate in pumped groundwater used for crop irrigation could be used to reduce inputs of commercial fertilizer and decrease N leaching to aquifers. Effectively managing N pollution also will require an understanding of the complex factors that control the N balance, including targeted regulations, individual human behavior, land-use conversion, and other ecosystem management practices that affect the N balance. These sometimes countervailing factors are illustrated with several scenarios of wastewater treatment technology and population growth in the Phoenix area. Management of N eventually must be coupled to management of other elements, notably carbon, phosphorus, and salts. We postulate that an ecosystem framework for pollution management will result in strategies that are more effective, fairer, and less expensive than current approaches.

## INTRODUCTION

Pollution control efforts in the United States have led to widespread reduction of xenobiotic organic compounds, lead and other metals, oxygen-consuming wastes, fecal coliforms and many other pollutants[1,2]. However, nitrogen pollution remains ubiquitous and largely unabated, posing a threat to humans through direct health effects and by impairment of ecosystem functioning[3,4]. Effective strategies for nitrogen management have failed in large part because they have been developed piecemeal, lacking an ecosystem framework.

This paper examines management of nitrogen cycling in the Central Arizona-Phoenix (CAP) ecosystem, a large metropolitan area surrounded by agricultural land and desert (Fig. 1). Key attributes of the ecosystem are described in Table 1.

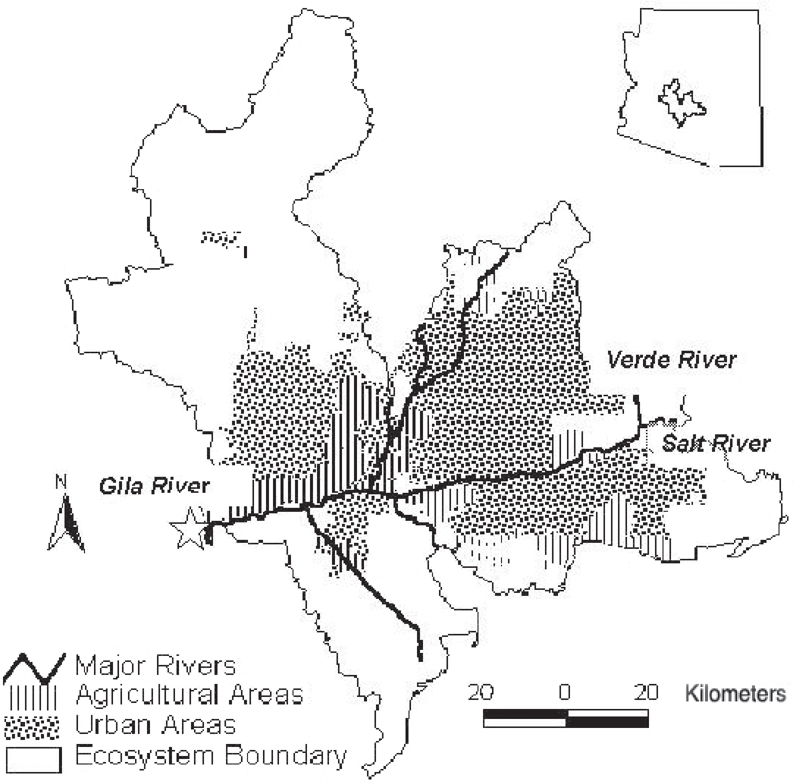

**FIGURE 1.** Map of the CAP-LTER watershed. The data in Fig. 2 was collected at the Gila River outflow, marked with a star (☆).

**TABLE 1**
**Key Attributes of the Central Arizona-Phoenix Ecosystem**

| | |
|---|---|
| Watershed area, $km^2$ | 12,384 |
| Precipitation, cm/year | 18 |
| Population, 1975, million | 1.3 |
| Urbanized area, % of watershed | 13 |
| Agricultural land, % of watershed | 10 |
| Desert, % of watershed | 77 |
| Ecosystem N input, Gg/year | 98.4 |
| Total ecosystem N output, Gg/year | 77.5 |
| Agricultural products, Gg/year | 2.5 |
| Export via Gila River, Gg/year | 2.6 |
| Export via atmosphere (inc. $N_2$ from dentrification),Gg/year | 2.6 |
| Accumulation, calculated by difference between inputs and outputs Gg/year | 20.9 |
| Accumulation, calculated by sum of fluxes to landfills and subsurface, Gg/year | 17.1 |

Data from Baker et al.[5].

In this paper we use a detailed nitrogen balance developed for the CAP ecosystem[5] to illustrate how multicompartment mass balances can be used to evaluate management practices, using the subsurface subsystem to illustrate how groundwater nitrate pollution could be largely controlled using "information engineering." We then examine how countervailing forces, in this case improvements in wastewater treatment and population growth, have altered N inputs to the ecosystem. Finally, we discuss the need to link elemental cycles in order to gain sufficient knowledge to effectively manage the biogeochemical cycles of cities.

## THE NITROGEN PROBLEM IN PHOENIX

Nitrogen pollution has persisted and grown worse in the Phoenix area over the past 30 years despite increasingly stringent regulatory measures to control it. For example, regulations now require major wastewater treatment plants in the Phoenix area to remove nitrogen from their effluents; at the same time, national regulations on automobile emissions (g N/mile) have become increasingly stringent. These measures have had little positive impact on nitrogen pollution of surface and groundwater. The use of nitrogen fertilizer increased soon after World War II (Fig. 2A). Nitrate levels in groundwater have increased steadily since the 1970s, with mean concentrations increasing from 7 to 8 mg $NO_3^-$-N/L to ~13 mg/L in the mid-1990s, then declining in the late 1990s (Fig. 2B). The increase tracks the escalating fertilizer use, with a lag time of 10–20 years, about the time it takes for water recharged at the surface to reach the aquifers. We cannot currently explain the rapid drop in groundwater nitrate concentrations since 1995, but mean levels remain ~10 mg $NO_3^-$-N, well above background levels. For reference, four wells located in undeveloped desert areas all have concentrations <5 mg $NO_3^-$-N/L.

Nitrogen concentrations in the Gila River near Gila Bend, the riverine outlet for the watershed, have remained nearly constant over the past 30 years (Fig. 3). Average and standard deviations (in mg $NO_3^-$-N/L) were 11.8 $\pm$ 4.9 (1963–1967), 12.3 $\pm$ 6.1 (1967–1976), 12.4 $\pm$ 5.1 (1977–1986), and 11.4 $\pm$ 4.0 (1987–2000). In summary, nitrogen contamination of surface and groundwater in the Phoenix ecosystem has not been controlled. While the situation in the Gila River has remained stable, groundwater has generally become more polluted, not less, over the past 30 years.

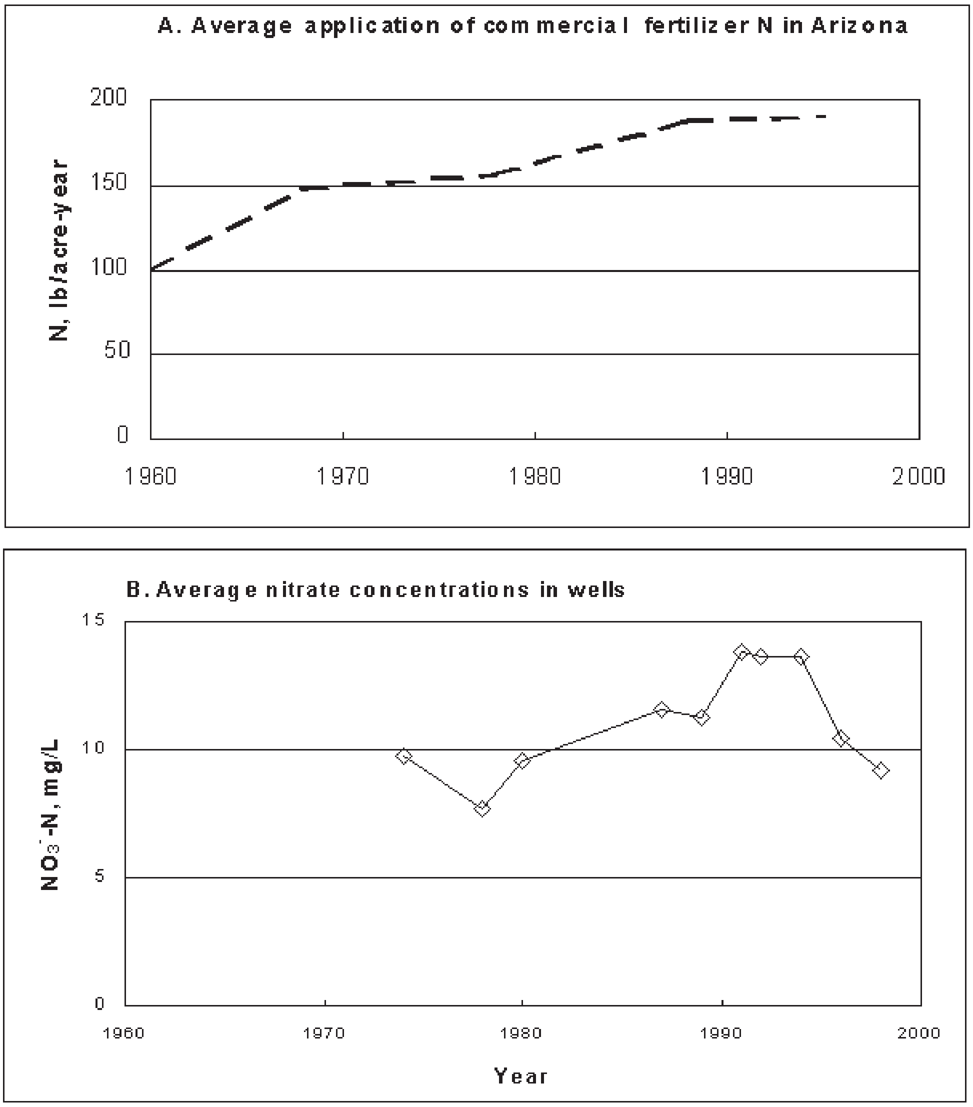

**FIGURE 2.** (A) Application of commercial N fertilizer in Arizona[6], and (B) average (krigged) groundwater nitrate concentrations in the CAP ecosystem.

## MASS BALANCE MODEL

Our N model for the CAP ecosystem includes nine subsystems (near-surface atmosphere, human food, urban landscapes, pets, wastewater, cropland, dairy, subsurface, and landfills) with 35 routes to transfer N to and from the ecosystem and among subsystems. Briefly, about 90% of the nitrogen input to the system is mediated by humans, either deliberately (e.g., importation of human and animal food) or inadvertently (e.g., fixation of $N_2$ by combustion processes; see Table 2). About 17–21% of the N input accumulates within the ecosystem. Most N export occurs by denitrification and export of $NO_x$ and ammonia. Less than 3% is exported via the Gila River, a reflection of the arid climate and intensive water conservation measures that promote volatilization, denitrification, and N accumulation, while minimizing riverine export[5].

### Utilizing the N in Pumped Groundwater: Case Study of Information Engineering

The ecosystem N balance is useful because it reveals where the main fluxes and storage of N occur in the system, thus providing an objective basis for targeting N pollution control strategies. As an example, consider the groundwater subsystem (Table 2). Inputs to the subsystem include septic tank leacheate, leaching from crops and urban landscapes, direct groundwater recharge using treated municipal wastewater. Currently, about 7.7 Gg N/ accumulates in the subsurface subsystem, a calculation that is at least qualitatively supported by the observed increase in groundwater nitrate concentrations (Fig. 2).

Of particular interest is the fact that a considerable amount of N (12.2 Gg/year) is pumped upward in groundwater. Most of the groundwater is used for irrigation of crops, adding 9.9 Gg N/ year to cropland (the rest of the pumped groundwater is used for municipal water supply). In some areas, the nitrate in groundwater would alone be sufficient to fertilize crops, with no addition of manure or commercial fertilizer. At the median groundwater concentration of 10 mg $NO_3^-$-N/L, irrigation with 1.5 m/year to crops (a typical irrigation rate) would supply 150 kg N/ha/year to the crop, sufficient or nearly sufficient to fertilize most crops[6]. Most farmers are unaware of the nitrogen fertilization potential of their irrigation water. Only about half of Arizona cotton farmers measure soil or petiole N[7]. The other half are not detecting oversupply of N. This is presumably true for other crops also.

The information provided by the subsurface N balance points to a novel approach for reducing groundwater N contamination: reduce the amount of commercial fertilizer to effectively utilize the N supplied by irrigation water. By reducing the oversupply of N, leaching of N to the subsurface would decline, eventually reducing groundwater nitrate levels. Two-thirds of commercial fertilizer application to crops could be eliminated if nitrate in pumped groundwater were utilized as fertilizer, while still providing recommended levels of N for crops. Leaching of nitrate to the subsurface would be decreased by 40%, reducing N accumulation in the subsurface by approximately two thirds (from 7.7 Gg/year to 2.7 Gg/year; see Table 2).

The scheme is complicated somewhat by the fact that the distribution of nitrate provided by irrigation water is far from uniform. Some farms use well water directly, but more commonly, groundwater is pumped into canals, where it is blended with varying amounts of surface water before being distributed to farms.

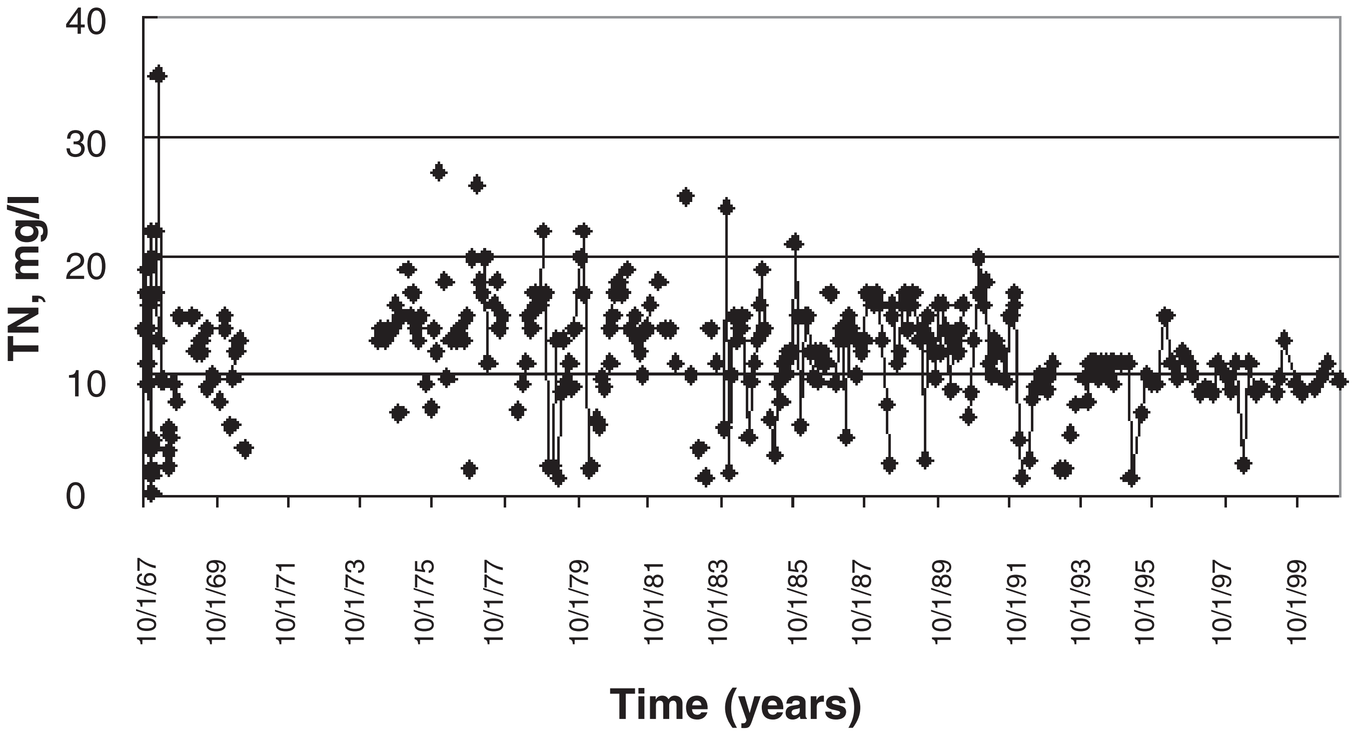

**FIGURE 3.** Total N in the Gila River as it exits the CAP ecosystem, 1967–2000 (U.S. Geological Survey).

**TABLE 2**
**Nitrogen Balance for the Subsurface Subsystem of the CAP Ecosystem***

| Component | Current flux, Gg N/year | Adjusted flux, using N in irrigation water to decrease commercial fertilizer application, Gg N/year |
|---|---|---|
| Major inputs to fertilized cropland | | |
| Commercial fertilizer | 14.0 | 4.1 |
| Alfalfa fixation | 7.5 | 7.5 |
| Manure application to land | 7.9 | 7.9 |
| N added by irrigation with groundwater | 9.9 | 9.9 |
| Other inputs (wastewater, biosolids, atm. dep.) | 4.6 | 4.6 |
| Total inputs | 43.9 | 34.0 |
| N removed by crop harvest | 17.7 | 17.7 |
| Loss to river | 1.7 | 1.7 |
| Leaching | | |
| Cropland | 12.3 | 7.3 |
| Leaching from urban landscapes | 6.2 | 6.2 |
| Leaching from septic tanks | 1.3 | 1.3 |
| Wastewater recharge | 0.1 | 0.1 |
| **Total inputs to subsurface subsystem** | **19.9** | **14.9** |
| **N pumped upward in groundwater** | **12.2** | **12.2** |
| **Accumulation in subsurface (inputs-pumping)** | **7.7** | **2.7** |

* The current N balance is shown in the left column. Changes that would result by accounting for nitrate in irrigation water as part of the farmer's fertilizer application program is shown in the right column. Excess N = total N inputs crop harvest loss to river; leaching was assumed to be 1/2 of excess N. Because current N inputs greatly exceed crop requirement, we assumed that crop harvest would not change with reduction of N input. Other inputs to the subsurface subsystem (leaching from urban landscapes and septic tanks; wastewater recharge) were unchanged.

Adapted from Baker et al.[5].

To complicate the matter further, the mix of well water and surface water changes over time, as wells are turned on and off to meet irrigation requirements. A decade ago, this would have presented an insurmountable problem for a nitrogen management strategy because it would have been nearly impossible to provide farmers with the information they need to reduce commercial fertilizer applications with confidence. Today's technologies can readily overcome the problem of temporal variability and spatial patchiness. A system of nitrate probes could be installed at key locations in canals, with data transmitted to a data processing center. Real-time data on nitrate concentrations would be converted to a form of information more useful to farmers: lb N applied per inch of irrigation water at the point of delivery to his or her fields. Farmers would receive this information via Web linkages and could track N applied via irrigation water over the season, adjusting inputs of commercial fertilizer accordingly. Farmers would benefit from reduced fertilizer costs (although fertilizer costs are a minor part of overall farm costs in Arizona), but the main benefit would be enhanced production of cotton, the primary cash crop, which suffers from overapplication of N.

This example of information engineering would be vastly cheaper in controlling N pollution to the ecosystem than conventional hard engineering approaches. Even a minor increase in the value of harvested crops would easily offset the cost of installing the monitoring network.

## RETROSPECTIVE ANALYSIS

Changes in N pollution are affected by many factors. The most obvious are deliberate regulations intended to reduce N pollution, such as the imposition of limits on N concentration in the effluent from wastewater treatment plants. However, individual human behaviors (e.g., choices in transportation, landscaping, and food consumption), other ecosystem management practices (e.g., water conservation or flood control measures), human population growth, and land-use conversion (e.g. agriculture to urban) are important factors that influence the N cycle of human ecosystems. Successful management of N pollution requires an understanding of all of these factors. Over time, these factors may work in countervailing directions.

The effect of these countervailing forces in altering the N balance over time is illustrated with the wastewater nitrogen balance for the CAP ecosystem from 1975 to 1995. Over this period, several regulatory measures have been implemented to reduce inputs of N from wastewater treatment plants to surface waters. Because of these regulations, 88% of the wastewater in the ecosystem now undergoes advanced treatment for N removal (biological nitrification-denitrification, or NDN), with an average N removal efficiency of 86%. Non-NDN plants have 45% N removal efficiency; consequently the average nitrogen removal for all treatment plants is 81%[8]. During the same period, the population served by municipal wastewater treatment plants increased from 72 to >90%, as homes that once used cesspools or septic tanks were hooked up to expanding sanitary sewer systems[9,10]. The population also nearly doubled over this period, from 1.3 million to 2.5 million. These two changes resulted in an increase in N entering wastewater treatment plants (Table 3). These engineering and population changes can be incorporated into scenarios to illustrate the effect of countervailing trends. In the worse-case scenario (Scenario 1), an increase in population with no change in wastewater treatment practice would have resulted in a 102% increase in the amount of N entering the Gila River and the subsurface subsystem. If the population had remained stable as treatment plants incorporated NDN (Scenario 2), N output from wastewater treatment plants would have declined by 40% (Table 3). In the actual situation (Scenario 3), population increase accompanied by incorporation of NDN resulted in a 13% decrease in N loading from wastewater treatment plants. If the incorporation of NDN had been accompanied by complete elimination of septic tanks (by hooking homes up to sanitary sewers) (Scenario 4), N input to river and subsurface would have declined by 34%, compared to 1975. In summary, the population increase largely offset the effect of switching to NDN, at least with respect to wastewater inputs to the environment.

Development of more complete historical scenarios are underway, and it should be possible to create a fairly realistic model of how the N cycle has changed over the past quarter century (reconstructions prior to the mid-1970s may be data-limited). It should also be possible to model the impact of various changes (human behaviors, regulations, etc.) separately and in various combinations to better understand how the N cycle would have changed with alternative histories. In doing so, we will gain not only a historical perspective on why N control policies have been unsuccessful, but also knowledge that can be used to manage the N cycle more successfully in the future.

## Coupled Biogeochemical Systems

Finally, as we move toward ecosystem-level management of human ecosystems, we must couple cycles of major elements — mainly carbon, nitrogen, phosphorus, water, and, in arid regions, salts — to develop holistic strategies for managing biogeochemical cycles. In the CAP ecosystem, for example, about 70% of the salt entering the ecosystem is retained, with much of the salt accumulating in the subsurface environment, resulting in a median TDS for groundwater in the ecosystem of 1000 mg/L and concentrations in some wells >3000 mg/L[12]. Accumulation of salts will probably continue in the absence of deliberate management. Because both salts (primarily major ions) and nitrate are soluble, management of N will inextricably be linked to management of salts. Management of nitrogen is also inextricably linked to

**TABLE 3**
**Four Scenarios Illustrating the Relative Effect of Engineering Changes and Population Growth in CAP Ecosystem**

| | Raw wastewater, Gg N/year | % treated in WWTPs | Gg N/year from WWTPs | Gg N/year leached (septic tanks and cesspools) | N entering the Gila River and subsurface, Gg N/year | Change since 1975, % |
|---|---|---|---|---|---|---|
| 1975 (baseline) | 7.2 | 72 | 2.9 | 1.8 | 4.7 | — |
| 1995 population, with 1975 treatment (scenario 1) | 16.5 | 72 | 8.3 | 1.2 | 9.5 | +102% |
| 1995 treatment, with 1975 population (scenario 2) | 7.2 | 92 | 1.0 | 1.8 | 2.8 | –40% |
| 1995 (actual) (scenario 3) | 16.5 | 92 | 2.9 | 1.2 | 4.1 | –13% |
| 1995, with no septic tanks (scenario 4) | 16.5 | 100 | 3.1 | 0 | 3.1 | –34% |

Data from References [8,9,10,11].

carbon, for example in combustion processes, denitrification, and animal nutrition. We currently have little understanding of coupled biogeochemical cycles in human ecosystems; developing this knowledge is a major research need for the next decade. Ultimately, it should be possible to develop a dynamic, multiconstituent, spatially explicit model that could be used to manage biogeochemical cycles in much the same way we now use models to simulate urban hydrology and transportation networks.

## CONCLUSIONS

Our understanding of biogeochemical cycles of human ecosystems is in an early state of development but can be expected to accelerate. The CAP N mass balance was used to demonstrate the potential for developing novel management practices with a case study demonstrating the potential for utilizing groundwater nitrate to partially replace commercial fertilizer. The model was also used to show how countervailing forces, in this case improved wastewater treatment and population growth, can limit the success of narrowly focused policies. We postulate that developing a better knowledge of biogeochemical cycles in human ecosystems could be used to develop pollution control practices that are more effective, fairer, and less expensive than current practices.

| | | |
|---|---|---|
| Received: | July | 13, 2001 |
| Revised: | September | 12, 2001 |
| Accepted: | September | 12, 2001 |
| Published: | October | 9, 2001 |
